# Natural Polyphenols as Immunomodulators to Rescue Immune Response Homeostasis: Quercetin as a Research Model against Severe COVID-19

**DOI:** 10.3390/molecules26195803

**Published:** 2021-09-25

**Authors:** Roberta Bernini, Francesca Velotti

**Affiliations:** 1 Department of Agriculture and Forest Sciences (DAFNE), University of Tuscia, Via S. Camillo de Lellis, 01100 Viterbo, Italy; 2 Department of Ecological and Biological Sciences (DEB), University of Tuscia, Largo dell’Università, 01100 Viterbo, Italy

**Keywords:** COVID-19, SARS-CoV-2, polyphenols, quercetin, immunomodulation, inflammation, cytokines, autophagy, macrophages, lymphocytes

## Abstract

The COVID-19 pandemic is caused by SARS-CoV-2 and is leading to the worst health crisis of this century. It emerged in China during late 2019 and rapidly spread all over the world, producing a broad spectrum of clinical disease severity, ranging from asymptomatic infection to death (4.3 million victims so far). Consequently, the scientific research is devoted to investigating the mechanisms of COVID-19 pathogenesis to both identify specific therapeutic drugs and develop vaccines. Although immunological mechanisms driving COVID-19 pathogenesis are still largely unknown, new understanding has emerged about the innate and adaptive immune responses elicited in SARS-CoV-2 infection, which are mainly focused on the dysregulated inflammatory response in severe COVID-19. Polyphenols are naturally occurring products with immunomodulatory activity, playing a relevant role in reducing inflammation and preventing the onset of serious chronic diseases. Mainly based on data collected before the appearance of SARS-CoV-2, polyphenols have been recently suggested as promising agents to fight COVID-19, and some clinical trials have already been approved with polyphenols to treat COVID-19. The aim of this review is to analyze and discuss the in vitro and in vivo research on the immunomodulatory activity of quercetin as a research model of polyphenols, focusing on research that addresses issues related to the dysregulated immune response in severe COVID-19. From this analysis, it emerges that although encouraging data are present, they are still insufficient to recommend polyphenols as potential immunomodulatory agents against COVID-19.

## 1. Introduction

The coronavirus disease 2019 (COVID-19) is an acute infectious disease caused by a novel β-coronavirus known as severe acute respiratory syndrome-coronavirus 2 (SARS-CoV-2) [1]. It results in a broad spectrum of clinical disease courses, ranging from asymptomatic and moderate courses (in the majority of the cases) to severe disease (in approximately 10–20% of symptomatic patients) characterized by severe pneumonia. Indeed, the leading cause of COVID-19 mortality is the respiratory failure due to acute lung injury (ALI) and its more severe form, acute respiratory distress syndrome (ARDS), which is associated with acute and severe inflammation in the lungs and sometimes matched by the failure of several other organs, including the kidneys, heart, and nervous system [2]. Although a certain number of vaccines, mostly preventing severe COVID-19, are currently available, there is no effective therapy for COVID-19, disease progression, and mortality.

The pathogenesis of COVID-19 is complex and multifactorial. Although the mechanisms underlying COVID-19 severity remain unclear, a central role of the host immune system in successful viral clearance or progression to severe and fatal disease has been established [3]. Indeed, dysregulation of the immune response homeostasis, concerning both the innate [4] and the adaptive [5] immunity, discriminates disease severity.

Although the pathobiology of immune dysregulation remains incompletely understood, in this review, we highlight the major immunological biomarkers of severe COVID-19 in order to assess whether polyphenols, a family of natural compounds widely diffused in the plant world and in commonly consumed vegetables and fruits [6], can affect these biomarkers and thus can be potentially used as immunomodulators to prevent and/or cure severe COVID-19. For this purpose, we critically analyze and discuss the in vitro and in vivo research on the immunomodulatory activity of quercetin, as a research model of polyphenols, showing some specific critical issues that have not yet been considered and have to be explored in future research before assuming the potential effectiveness of polyphenols against COVID-19.

## 2. COVID-19 and Dysregulated Immune Response in Severe Disease

### 2.1. Dysregulated Innate Immune Response in Severe COVID-19

**Figure 1:**
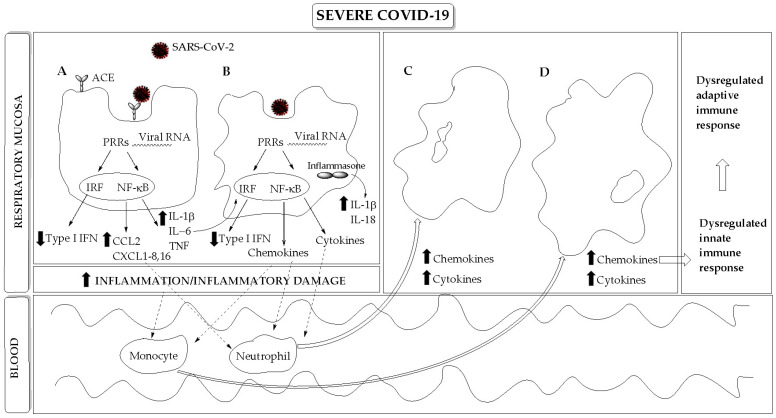
Immune response dysregulation induced by SARS-CoV-2 in severe COVID-19. Legend: (**A**) Respiratory epithelial cells; (**B**) Resident macrophages; (**C**) Blood-recruited neutrophils; (**D**) Monocyte-derived macrophages. Abbreviations: ACE = Angiotensin Converting Enzyme; PRRs = Pattern Recognition Receptors; NF-κB = Nuclear Factor-κB; IRF = Interferon Regulatory Factor; IFN = Interferon; IL = Interleukin; TNF = Tumor Necrosis Factor; CCL = C–C motif Chemokine Ligand; CXCL = C–X–C motif Chemokine Ligand.

The decision on the effectiveness of the immune response against SARS-CoV-2 seems to be made very early after virus infection. Since the innate immune system is the very early line of defense against pathogens, the innate immune response seems to play a central role in this decision (Figure 1). Innate immunity is composed of two categories of defense, the physical/chemical barriers, consisting of epithelial cells separating the organism from the external environment, and the innate immune cells, consisting of tissue resident and peripheral blood recruited immune cells.

The major entry site of SARS-CoV-2 is the respiratory epithelial barrier, where the virus can enter the upper and lower respiratory tract, infecting nasal and alveolar epithelial cells, respectively. The virus can also enter the cornea, esophagus, ileum, colon, gallbladder, and common bile duct. Little evidence is present that innate immune cells are a target of virus infection; thus, it is not clear whether the virus infects macrophages or enters these cells via phagocytosis [4]. Notably, early steps of antiviral immune responses mediated by both infected barrier epithelial cells and activated innate immune cells play an important role in the initial viral infection, spread, and clearance, determining the severity of COVID-19. Barrier mucosal epithelial cells, such as respiratory epithelial cells, recognize SARS-CoV-2 via intracellular pattern recognition receptors (PRRs) (Figure 1), very likely via endosomal Toll-like receptor (TLR)3 and TLR7, and via cytoplasmic retinoic acid-inducible receptor (RIG)-1 and melanoma differentiation-associated protein (MDA)5. These receptors are linked to signaling pathways, activating the anti-viral type I interferon (IFN) response program and the nuclear factor (NF)-κB-mediated inflammatory response (Figure 1). Both the type I IFN and NF-κB-mediated inflammatory responses must be finely tuned, in that their overactivation or underactivation are harmful to the host.

In SARS-Cov-2 infection, researchers have found the early inhibition of the anti-viral type I IFN response, which is associated with the boosting of inflammatory mediator production, including cytokines such as interleukin (IL)1β, IL6, tumor necrosis factor (TNF), and neutrophil-recruiting chemokines, such as C–C motif chemokine ligand (CCL)20 and C–X–C motif chemokine ligand (CXCL)1, CXCL2, CXCL3, CXCL5, CXCL6, CXCL8, and CXCL16 [7] (Figure 1, Table 1). Therefore, a current model of severe COVID-19 suggests that firstSARS-CoV-2 infects lung alveolar epithelial cells, inducing an inflammatory condition characterized by the exacerbated production of inflammatory cytokines and chemokines, which is associated with alveolar epithelial cell death. Then, inflammatory cytokines activate resident macrophages, such as alveolar macrophages, which are a major source of cytokines and chemokines, leading to further tissue accumulation of NF-κB-dependent inflammatory molecules, which hence promote lung pathogenic infiltration of blood recruited neutrophils and monocytes/macrophages (Figure 1). Infiltrating cells further increase the level of inflammatory mediators in the lung, bronchoalveolar lavage fluid (BALF) (e.g., CCL2, CCL3, CCL4, and CXCL10), and peripheral blood (e.g., IL-1, IFNγ, IL-17, TNF, IP-10, MCP-1, G-CSF, GM-CSF, IL-1RA, CCL2, CCL3, CCL5, CCL8, CXCL2, CXCL8, CXCL9, and CXCL16) [7,8], leading to a serious systemic dysregulation of immune homeostasis. Finally, the large amounts of cytokines hyperactivate signal transducer and activated transcriptions (STATs) signaling pathways, thus contributing to further exacerbate inflammation [9].

The interaction between SARS-CoV-2 and TLR3 also triggers the transcription of the NRL family pyrin domain containing 3 (NLRP3) gene, which, together with other stress-induced cell responses, contributes to the activation of the NLRP3 inflammasome [10] (Figure 1). The NLRP3 inflammasome is an intracellular molecular platform that promotes inflammation via the activation of key inflammatory cytokines such as IL-1β and IL-18 (Figure 1), stimulating adaptive immune responses against pathogens. However, the aberrant activation of the NLRP3 inflammasome exacerbates inflammation, being involved in the pathogenesis of several inflammatory disorders and in septic shock [11]. Of note, the extent of NLRP3 inflammasome activation, correlating with the excessive IL-1β production, also correlates with COVID-19 severity (Table 1) and poor clinical outcome [10].

Another emerging hallmark of severe COVID-19 is the inhibition by SARS-CoV-2 of the cell host-clearing systems, such as the proteasome/immunoproteasome and the autophagy pathways [12,13] (Table 1), interfering not only with virus replication in infected cells but also with cytokine-mediated inflammation at both cellular and multisystem levels. SARS-CoV-2 hijacks both the immunoproteasome-dependent processing of viral antigen peptides, impairing the subsequent activation of cytotoxic T-lymphocyte (CTL)-mediated adaptive immune response via MHC-I presentation [12], and autophagy, which is a key pathway to balancing antimicrobial immune responses by inducing viral clearance (xenophagy), T helper (Th)-cell-dependent adaptive immune responses, and immunoglobulin production, meanwhile preventing excessive inflammation and immune stimulation [13].

Although barrier epithelial cells and macrophages are among the main innate immune cells involved in the exacerbated inflammatory reactions involved in tissue damage (Figure 1), especially in lung injury, other innate immune cells contribute to COVID-19 severity. Among them, neutrophils, the first inflammatory cells migrating to the inflammatory tissue, are abundant in SARS-CoV-2 infected tissues (Figure 1), in BALF and in peripheral blood [4]. An increase in circulating neutrophils (neutrophilia) versus a decrease in lymphocytes (lymphopenia) has been established as a hallmark of severe COVID-19 (Table 1). Moreover, neutrophils show expression profiles associated with activation, degranulation, and neutrophil extracellular trap (NET) formation (i.e., ejection of DNA strands to trap pathogens) [14]. Of note, neutrophilia and the release of NETs are characteristic of ARDS, and NETs seem also to be involved in thrombotic complications and coagulopathy frequently found in severe COVID-19 (Table 1). In addition, elevated levels of eosinophils (eosinophilia) are also found in severe COVID-19 and appear to contribute to inflammation [9] (Table 1), whereas basophils are reduced [4]. Finally, a decreased number of circulating NK cells, anti-viral lymphocytes of the innate immunity, have been observed in severe COVID-19; NK cells also show an increased surface expression of NKG2A inhibitory receptor associated with a functionally exhausted phenotype [15] (Table 1).

In conclusion, evidence suggests that severe COVID-19 is characterized by a dysregulated innate inflammatory response, especially in the lung, which is associated with the excessive and uncontrolled release of inflammatory cytokines referred to as a “cytokine storm”, which leads to ALI/ARDS, pulmonary edema, alveolar epithelial cell death, and finally, vascular damage and multi-organ failure [2,3,9].

The massive elevation of cytokines in severe COVID-19 is also accompanied by elevated circulating levels of innate soluble receptors, such as C-reactive protein (CRP), pentraxins, and complement fragments [9] (Table 1).

### 2.2. Dysregulated Adaptive Immune Response in Severe COVID-19

Severely ill patients display a compromised adaptive immune system, which is characterized by serious lymphopenia [5] (Table 1). It is currently unclear whether lymphopenia is caused by an inhibition of lymphocyte proliferation (induced by an immunosuppressive microenvironment) or by lymphocyte apoptosis (induced by inflammation and/or SARS-CoV-2 infection).

Concerning T lymphocytes, T-cell exhaustion has been observed in severe COVID-19 [15] (Table 1). Another pathological event, which is associated with a worse outcome of the disease, is the imbalance between Th17 and Treg lymphocytes [16,17] (Table 1). Evidence suggests that the increased number of Th17 cells play an important role in COVID-19 progression not only by activating the cytokine cascade (through secretion of the inflammatory cytokine IL-17, which promotes tissue neutrophil recruitment) but also by inducing Th2 responses, inhibiting Th1 differentiation, and suppressing Treg cells [17]. A decreased number and function of Treg cells in severe COVID-19 leads to impaired regulation of inflammation and immune response homeostasis, thus contributing to the uncontrolled inflammatory status.

Concerning B lymhocytes and their antibody response, it has been observed that the delayed production of SARS-CoV-2 neutralizing antibodies correlates with severe and fatal COVID-19 [18] (Table 1).

### 2.3. Age and Dysregulated Immune Response in Severe COVID-19

High risk factors for COVID-19 severity and lethality are age and chronic inflammatory diseases, including diabetes as well as cardiovascular and neurodegenerative diseases.

Age is often associated with a chronic, sterile inflammatory state denoted inflammaging [19], which shares inflammatory critical biomarkers with severe COVID-19 (Table 1). Indeed, older individuals typically produce weaker type I IFN responses upon viral infection. In addition, the aged innate immune system increases myelopoiesis, resulting in an increased number of neutrophils and monocytes in the circulation as well as of inflammatory macrophages in tissues [4]. Moreover, T lymphocytes are decreased, and thymic involution is the most critical contributor of reduction in naïve T cells with age. Thus, the neutrophil-to-lymphocyte ratio positively correlates with advancing aging. Finally, myeloid cells express an inflammatory profile associated with NLRP3 activation and IL-6, IL-12, and IL-1β secretion, leading to elevated levels of inflammatory mediators in peripheral blood, including IL-6, TNF, CXCL8, CCL2, and CRP.

A recent study illustrated that age has an impact on inflammatory mediators present in severe COVID-19 [20]. It can be hypothesized that the innate immune response in COVID-19 exacerbates the already existing misbalance of the immune system present in the elderly toward a further pro-inflammatory and dysfunctional state. Based on the identification of large numbers of dysfunctional immune cells, it has been postulated that the higher COVID-19 severity in aged individuals is caused by dysregulated rather than impaired host innate immunity [4].

Finally, it should be pointed out that since inflammaging is a significant risk factor for age-related inflammatory conditions such as diabetes, cancer, cardiovascular, autoimmune, and Alzheimer’s diseases, age is likely to be one of the most important risk factors for severe COVID-19 [2].

## 3. Polyphenols

Polyphenols represent the most abundant and widespread class of plant secondary metabolites. According to the first definition proposed in 1962 by Bate-Smith and Swain [21], they are ‘‘water soluble compounds having molecular weights between 500 and 3000 (Da) and, besides giving the usual phenolic reactions, they have special properties such as the ability to precipitate alkaloids, gelatin and other proteins from solution’’. A more recent definition states that “the term polyphenol should be used to define plant secondary metabolites derived exclusively from the shikimate derived phenylpropanoid and/or the polyketide pathway(s), featuring more than one phenolic ring and being devoid of any nitrogen-based functional group in their most basic structural expression’’ [22].

In nature, polyphenols occur primarily in glycosylated forms, and they are linked to one or more sugar residues (glucose, galactose, rhamnose, xylose, and arabinose) as well as esters, in particular methyl esters, and as prenylated and geranylated derivatives [23]. They are found in almost all food of plant origin (vegetables, cereals, legumes, fruits, nuts) and beverages (wine, cider, beer, tea). In each matrix, the content is influenced by several parameters as environmental conditions, genetic factors, germination, degree of ripeness, variety, processing, and storage [24].

**Figure 2:**
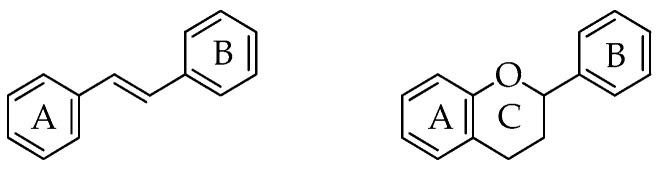
Basic skeleton of stilbenes and flavonoids.

To date, in the plant kingdom, we identified more than 8000 compounds with a wide range of structural variety ranging from simple phenols to highly polymerized compounds [23]. Among the different classes of polyphenols, particular attention is turned to stilbenes and flavonoids. The basic structure of stilbenes consists of two aromatic rings linked by a double bond, generally in (*E*) configuration, while the skeleton of flavonoids consists of 15 carbon atoms arranged in two rings (A e B) joined by a benzopyranic C ring (Figure 2). In both basic structures, one or more hydroxyl groups are linked to the aromatic rings. Hydroxyl groups are free, esterified, and/or linked to sugars.

**Scheme 1:**
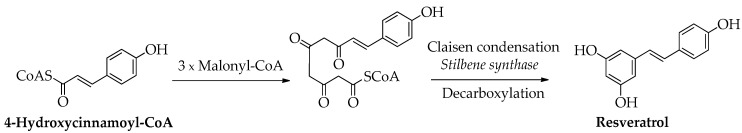
Biosynthesis of resveratrol.

Biogenetically, both stilbenes and flavonoids derive from 4-cinnamoyl-CoA and three malonyl-CoA units [25]. The resulting polyketide folds via Claisen or aldol cyclization, according to the enzyme that catalyzes the reaction. Stilbene synthase promotes the Claisen condensation, and after decarboxylation reaction, the resulting compound is resveratrol (Scheme 1). It is a stilbene found in grapes and wine as well as in other food products exhibiting antioxidant, anti-inflammatory, anti-platelet, and anticancer properties [26]. Enzymes such as methyltransferases, peroxidases, prenyltransferases, and glucosyltransferases catalyze chemical modifications of the stilbene backbone to afford a wide number of derivatives [27].

**Scheme 2:**
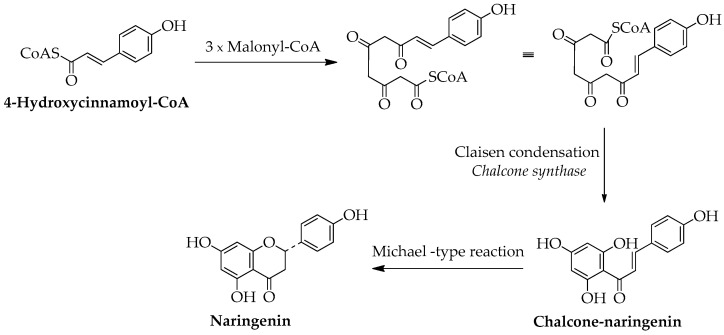
Biosynthesis of chalcone-naringenin and naringenin.

Chalcone synthase catalyzes the aldol condensation, producing naringenin-chalcone, which gives naringenin (a flavanone) via a Michael-type nucleophilic addition of a phenol group on to α,β-unsaturated ketone (Scheme 2). This reaction is enzyme-catalyzed and stereospecific, resulting in the formation of a single flavanone enantiomer [25].

**Scheme 3:**
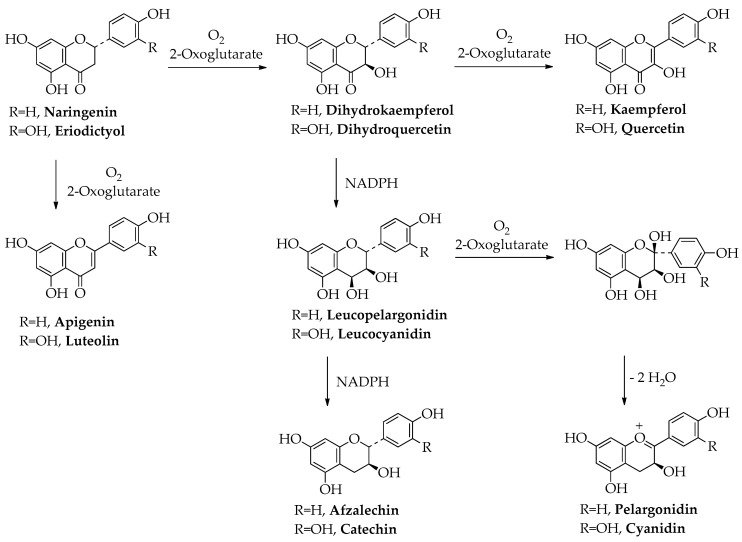
From flavanones to other flavonoids.

Flavanones as naringenin or eriodictyol give rise to a variety of structural modifications (Scheme 3) including flavones (e.g., apigenin, luteolin); dihydroflavonols and flavonols (e.g., dihydrokaempferol, dihydroquercetin and kaempferol, quercetin, respectively); flavandiols (e.g., leucoperlargonidin, leucocyanidin); flavan-3-ols (e.g., afzalechin, catechin); and anthocyanidins (e.g., pelargonidin and cyanidin) [25]. Even if colorless, flavones absorb strongly in the UV spectrum and are detectable by insects, aiding flower pollination; chalcones, flavonols, and anthocyanidins contribute to plant colors from yellow to red, blue, and violet.

Flavonoids constitute the largest group of plant phenolics with more than 5000 different compounds. Modifications to the hydroxylation patterns in the two aromatic rings as well as methylation and dimethylation reactions occur in nature, increasing the range of compounds. A high number of flavonoids are water-soluble glycosides. Generally, glycosylation occurs at the C-3, C-5, and C-7 positions and the sugars are mainly rhamnose, glucose, galactose, and arabinose [28].

In the Mediterranean area, considerable quantities of flavonoids are taken daily with the diet, and their beneficial effects on human health are widely demonstrated by the scientific literature [29].

In plant tissues, quercetin facilitates several physiological processes such as seed germination, pollen growth, photosynthesis, plant growth, and development [30]. It is found in several edible vegetables as onion, asparagus, and berries. Quercetin is a powerful anti-inflammatory and antioxidant compound, chelating metals, scavenging free radicals, and preventing the oxidation of low-density lipoprotein [31].

**Figure 3:**
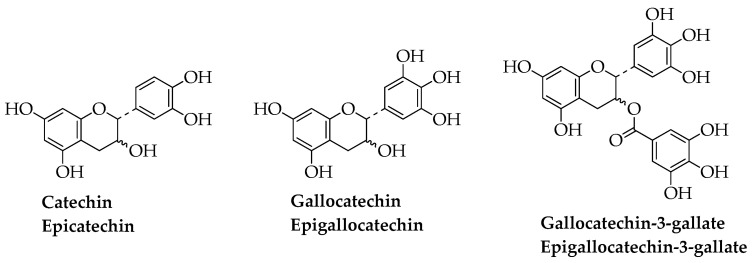
Catechin derivatives.

The principal source of catechin, epicatechin, gallocatechin, epigallocatechin, epicatechin-3-gallate, epigallocatechin-3-gallate, and epicatechin gallate (Figure 3) is green tea, and epigallocatechin-3-gallate is the major component [32]. Other sources are grapes, blackberries, apples, beans, almond, pistachios, cocoa-based products, and red wine [33].

Catechins constitute the starter units for the biosynthesis of proanthocyanidins (or condensed tannins), which are small polymers responsible for the astringency of foods and drinks. Legumes, nuts, grape seeds, pine bark extracts, and cereals such as sorghum and barley are rich sources of these compounds. They are responsible for the anti-inflammatory activity of natural extracts [34].

A second class of tannins are hydrolyzable tannins [35], including gallotannins and ellagitannins that are esters of gallic acid and hexahydroxydiphenic acid, respectively, with a polyol, which is generally glucose. These polymers can be found in all varieties of berries and their derivatives (such as juices, jams, and jellies), pecans, walnuts, brazil nuts, peanuts, pomegranate (fruit and juice) [36], and chestnut [37].

**Figure 4:**
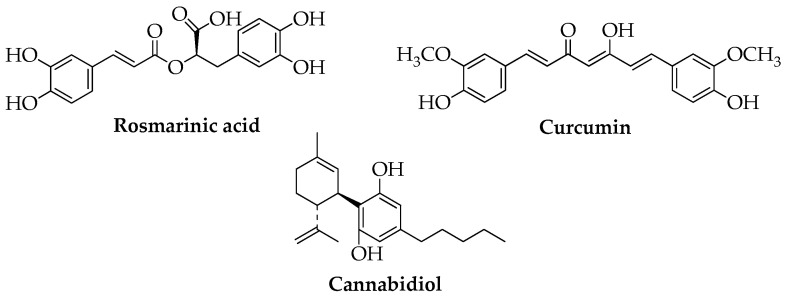
Chemical structure of rosmarinic acid, curcumin, and cannabidiol.

Other bioactive polyphenols are rosmarinic acid, curcumin, and cannabidiol (Figure 4). Rosmarinic acid is the ester of 3,4-dihydroxyphenyllactic acid and caffeic acid found in more than 30 families of plants including *Mentha piperita* L., *Melissa officinalis* L., *Rosmarinus officinalis* L., and *Salvia officinalis* L. According to the presence of two catecholic moieties, it shows a remarkable antioxidant, anti-inflammatory, and immunomodulatory activity but also the antiviral, neuroprotective, and anticancer properties are relevant [38]. Curcumin is a natural diphenolic compound, yellow colored, isolated from the rhizome of *Curcuma longa* L., where it is present in relatively high concentrations. Used both as a spice and dietary supplement, it shows cardioprotective, neuroprotective, antidiabetic, anti-inflammatory, and anticancer activity [39].

**Scheme 4:**
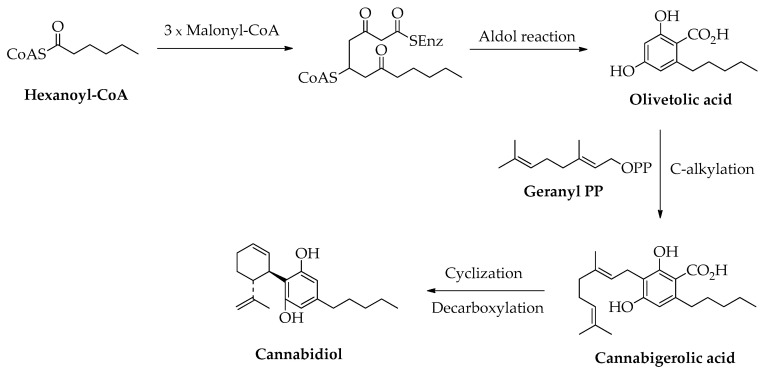
Biosynthesis of cannabidiol [25].

Cannabidiol belongs to the class of cannabinoids, which is a group of terpenophenolics found in *Cannabis sativa* L., a plant native to Central Asia that was later distributed in America and Europe. Chemically, it consists of a phenolic ring having a C5 alkyl chain and a cyclic monoterpene C10 unit. As depicted in Scheme 4, in the biosynthetic pathway [25], the precursor of the phenolic moiety is olivetolic acid obtained from aldol cyclization of the polyketide derived from hexanoate-CoA and three units of malonate-CoA. Cannabigerolic acid is the compound resulting after C-alkylation of olivetolic acid with geranyl diphosphate. The following cyclization of the monoterpene unit and decarboxylation reaction give cannabidiol. This non-psychoactive cannabinoid is useful in different therapeutic applications including treatment of cancer, Alzheimer’s disease, multiple sclerosis, glaucoma, pain, and inflammation [40].

### 3.1. Polyphenols as Immunomodulators Counteracting Dysregulated Immune Responses Related to Severe COVID-19

Polyphenols are powerful immunomodulatory, anti-inflammatory, and antiviral agents [29,41,42]. Not less important, polyphenols have an established role not only in determining one’s inflammatory status [43] but also in preventing aging [44], which is one of the main risk factors for severe COVID-19. The immunomodulatory activity of polyphenols has supported their experimental use as therapeutic tools in different acute and chronic inflammatory disorders, including ALI [45], cardiovascular diseases [46], obesity, diabetes [47,48], and cancer [49]. Therefore, considering all the beneficial properties of polyphenols in human health and diseases, currently, they have also been proposed to counteract the immune response dysregulation in COVID-19, which is the main life-treating condition in severe COVID-19 patients [50,51,52,53,54,55]. The use of polyphenols, such as cannabidiol, curcumin, epigallocatechin-3-gallate, resveratrol, and quercetin has been recently approved in clinical trials for COVID-19 prevention and/or therapy (Table 2).

Since quercetin is the most widely studied among polyphenols [52,53,56], in this review, we take quercetin as a model to examine the potential use of polyphenols as immunomodulators in COVID-19 treatment. Here, we reference the in vitro and in vivo immunomodulatory activity of quercetin potentially affecting the immunological biomarkers of severe COVID-19 (Table 1), while we refer to other reviews for its direct antiviral activity [57,58,59,60,61].

#### 3.1.1. Quercetin as Immunomodulator in In Vitro Inflammatory Cell Models

Although the immunomodulatory and anti-inflammatory activity of quercetin has been assessed in several different experimental cell type models [56,62,63,64], here, we examine the research focused on the activity of quercetin on immune cells and molecules mostly implicated in the pathogenesis of severe COVID-19 (Figure 1), including barrier respiratory epithelial cells, alveolar and monocyte-derived macrophages, neutrophils, T lymphocytes, as well as their cell functions, such as the production of inflammatory mediators (cytokines, chemokines, and soluble innate receptors) as well as the activation of the inflammasome and the autophagy pathways (Table 1).

Very few in vitro studies have been focused on the immunomodulatory effects of quercetin on the immune response mediated by barrier mucosal epithelial cells such as lung epithelial cell lines and, to the best of our knowledge, there is no study focused on the effect of quercetin on the immune response by lung epithelial cells undergoing viral infection. However, Geraets et al. [65] showed a dose-dependent inhibition by quercetin of IL-8 production in lipopolysaccharide (LPS) stimulated A549 human lung epithelial cells. Although LPS stimulation represents an experimental model of bacterial infection, this result is important, since IL-8 (CXCL-8) is a chemoattractant cytokine that specifically targets neutrophils, which are inflammatory cells implicated in COVID-19 severity (Table 1). The response of neutrophils to IL-8 is characterized by their migration to the inflammatory site, their activation, and their degranulation, resulting in the release of tissue-damaging proteases and mediators, as well as by the formation of NETs, causing host tissue damage. In another study, it has been found that quercetin inhibits the expression of the pro-inflammatory intercellular adhesion molecule-1 (ICAM-1) in IL-1β-primed A549 pulmonary epithelial cells through the mitogen-activated protein kinase (MAPK) pathway [66]. Then, Gunay et al. [67] showed that quercetin induced a significant decrease in IFN-γ, IL-1β, and TNF-α inflammatory cytokines, in benzo(a)pyrene exposed A549 alveolar epithelial cells, proposing the use of quercetin in the prevention and treatment of inflammatory lung diseases caused by smoking.

No in vitro experiments have been performed to investigate the capability of quercetin of modulating type I IFN production in virus infected barrier mucosal epithelial cells nor in the human bronchial epithelial cell line 16HBE, which is widely utilized as an experimental model for respiratory epithelial diseases and barrier function [68]. However, it has been recently hypothesized that quercetin, by its capability of inhibiting casein kinase 2, can restore the production of type I IFN in response to the interaction virus-RIG-I receptor, thus promoting the control of virus infections [69]. Moreover, some non-respiratory barrier epithelial cell models have been used to investigate the regulation of inflammasome and autophagy by quercetin. Indeed, it was found that quercetin exerts an anti-inflammatory preventive activity on the human colonic epithelial Caco-2 cell line upon *E. Coli* infection, which is due to the inhibition of NLRP3 inflammasome and the promotion of autophagy [70]. In addition, in a study performed on IFN-γ-primed keratinocytes infected by a double-stranded (ds) DNA virus, quercetin inhibited NLRP3 inflammasome activation via the inhibition of interferon inducible protein AIM2 and pro-caspase-1 expression, thus decreasing severe inflammation caused by virus-induced IL-18 production [71].

A paucity of research also exists on the in vitro immunomodulation by quercetin of the immune response mediated by lung-resident macrophages, suggesting a need for more experiments on alveolar macrophages, using human alveolar macrophage models and primary alveolar macrophages (e.g., from mice). However, an interesting study carried out by Yasui et al. [72] showed that quercetin inhibited TLR7-induced expression of TNF-α and IL-6 as well as NF-κB activation, both in mouse alveolar macrophages (AMJ2-C11 cells) and in mouse BALF cells, supporting the hypothesis that dietary supplementation with quercetin may have efficacy in the treatment of inflammation in respiratory viral infections. In another study, Takashima et al. [73] showed a marked suppression by quercetin of pro-inflammatory cytokine production by LPS-induced AMJ2-C11 alveolar macrophages.

Several investigations have been focused on the in vitro immunomodulatory activity of quercetin on inflammatory experimental models in rodent and human monocyte-derived macrophages. As early as 1999, Manjeet et al. [74] showed that quercetin significantly inhibited TNF-α production in LPS-stimulated murine RAW 264.7 macrophages in a dose and time-dependent manner. However, it is interesting to note that this inhibition was observed when quercetin was added prior or simultaneously to LPS addition, whereas it was not significant when quercetin was added after LPS, suggesting that quercetin might be able to maintain but not restore immune homeostasis in inflammatory responses. Then, Kim and Park [75], using a model of double-stranded (ds) RNA virus-induced macrophages, showed that quercetin significantly decreased the production of inflammatory mediators (IL-6, MCP-1, IP-10, RANTES, GM-CSF, G-CSF, TNF-α, LIF, LIX, and VEGF) and inflammatory signaling (STAT1 and STAT3) in RAW 264.7 macrophages. These data indicate that quercetin is capable of reducing the effects of viral inflammation through the inhibition of cytokines, chemokines, growth factors, and the STAT pathway [75]. Moreover, in LPS-stimulated RAW 264.7 cells, quercetin induced the suppression of IL-1β, IL-6, and TNFα production through the inhibition of signaling pathways such as extracellular signal-regulated kinase (ERK) and p38 MAPK as well as NF-κB [76,77]. Finally, quercetin decreased LPS-induced inflammation in RAW 264.7 cells via the inhibition of Src- and Syk-mediated phosphatidylinositol-3-kinase (PI3K)-(p85) tyrosine phosphorylation and subsequent TLR4/MyD88/PI3K complex formation, thus decreasing the activation of downstream signaling pathways [78]. In addition to murine macrophage models, human macrophage experimental models have also been used. In a study, quercetin prevented inflammation in the human macrophage differentiated cell line U937, inhibiting the basal expression of inflammatory genes (TNF-α, IL-6, IL-8, IL-1β, IFN-γ, and IP-10), as well as the level of phosphorylated c-Jun *N*-terminal kinase (JNK) and c-Jun, and causing IκBα degradation [79]. Then, Mendes et al. [80] showed that quercetin decreased TNF-α, IL-1β, and IL-6 production by human pre-polarized inflammatory M1 macrophages (LPS  +  IFN-γ pre-cultured THP-1 cells). Quercetin was also responsible for a marked reduction, in a dose-dependent manner, of the production of TNFα by normal peripheral blood mononuclear cells, and this effect was mediated through the modulation of NF-κB and IκB [81].

Studies are also present, indicating that the inhibition by quercetin of inflammatory cytokine production by macrophages is mediated by its capability to inhibit the NLRP3 inflammasome and promote autophagy [82,83,84]. Indeed, quercetin prevented the increased expression of IL-1β, IL-18, and caspase-1 through the inhibition of NLRP3 inflammasome activation in fructose-induced U937 and THP-1 human macrophages, thus preventing the exacerbation of inflammation [82]. In another study, quercetin significantly reduced IL-1β production by monosodium urate crystals-stimulated human THP-1 cells through NLRP3 inflammasome inhibition [83]. Finally, quercetin inhibited the formation of foam cells generated by ox-LDL-induced RAW264.7 cells and delayed a senescence phenotype by promoting autophagy, thus potentially suppressing atherosclerosis progression [84].

A certain number of investigations have also shown the immunomodulatory effect of quercetin on neutrophil recruitment, activation, degranulation, and NET formation [85,86,87,88]. Concerning neutrophil recruitment, Souto et al. [85] showed a dose-dependent inhibition by quercetin of the chemoattraction of human neutrophils induced by CXCL8, LTB(4), and fMLP, highlighting the possible usefulness of quercetin to diminish excessive neutrophil migration to tissues during inflammation. Then, concerning neutrophil activation and cytokine production, two studies suggest that quercetin prevents neutrophil-mediated inflammation and neutrophil-mediated inflammatory diseases. One study showed that pre-treatment with quercetin abrogated IL-6 production by LPS-stimulated human neutrophils [86]. The other study showed that pre-incubation with quercetin significantly inhibited TNF-α productions in phorbol 12-myristate 13-acetate (PMA)-stimulated human neutrophils [87]. Finally, concerning neutrophil degranulation, by analyzing elastase release by neutrophils, Kanashiro et al. [88] showed that quercetin is a potent inhibitor of neutrophil degranulation, supporting the use of quercetin as a potential therapeutic agent in the treatment of neutrophil-dependent inflammatory diseases.

Finally, contrasting results are present concerning the in vitro modulation by quercetin of T-cell mediated adaptive immune responses. In normal peripheral blood mononuclear cells, Nair et al. [89] showed that quercetin significantly induces the polarization of Th lymphocytes in Th-1 cells producing IFN-γ, downregulating the polarization of Th-2 producing IL-4 and suggesting an immunostimulatory anti-viral activity by quercetin through the regulation of Th-1 polarization. In contrast, the in vitro treatment of activated T cells with quercetin blocked IL-12-induced tyrosine phosphorylation of JAK2, TYK2, STAT3, and STAT4, resulting in a decrease in IL-12-induced T-cell proliferation and Th1 differentiation [90].

#### 3.1.2. Quercetin as Immunomodulator in In Vivo Inflammatory Animal Models

Since respiratory failure due to ALI/ARDS is associated with acute and severe inflammation in lungs, which characterizes severe COVID-19 and represents the leading cause of COVID-19 mortality, we first mention research focused on the in vivo immunomodulatory activity of quercetin in different ALI animal models. It has been reported that quercetin protects against different experimental rat and murine models of ALI, such as those induced by acid aspiration, radiation, cigarette smoke, and LPS, as shown by improved animal survival, lung edema, and lung histological changes [91]. It was found that the beneficial immunomodulatory effects of quercetin in different models of ALI is mostly associated to its anti-inflammatory activity, consisting of the induction of the decrease in peribronchial and alveolar inflammatory cell infiltration [92], the suppression of NF-κB and MAPK inflammatory signaling pathways [93], the reduction of inflammatory cells in BALF, TNF-α, and TGF-β1 concentrations in plasma, and inflammatory response in tissues [94]. It was reported that the preventive administration of quercetin exerted beneficial effects on experimental murine LPS-induced septic acute lung injury, since quercetin markedly rescued lethality, improved survival time, decreased lung pathological changes, and inhibited serum levels of TNF-α, IL-1β, and IL-6 while also increasing IL-10 levels and significantly reducing the NF-κB signaling pathway [73,95]. According to these data, Da Silva et al. [96] found that mice, receiving quercetin via orogastric gavage before exposure to cigarette smoke, demonstrated reduced leukocyte levels, histological pattern changes of pulmonary parenchyma, and lung function alterations, suggesting the preventive activity of quercetin on the inflammatory effects of cigarette smoke exposure. Of note, beneficial anti-inflammatory effects of quercetin have also been found in ALI caused by viral infections. In fact, quercetin reduced lung inflammation induced by rhinovirus-infected mice while inhibiting the accumulation of cytokines and chemokines (CXCL-1 and CXCL-2, TNF-α and CCL2), thus effectively mitigating rhinovirus-induced inflammatory exacerbation [57]. In addition, quercetin supplementation effectively prevented the rhinovirus-induced progression of lung disease in a mouse model of COPD, and this effect was associated with a reduction of accumulation of inflammatory neutrophils, macrophages, and T cells in the lungs [97]. Finally, quercetin caused increased levels of the anti-inflammatory cytokine IL-27 in influenza A-treated Madin–Darby canine kidney (MDCK) cells [98].

The beneficial immunomodulatory activity of quercetin has also been assessed in other animal models of acute and chronic inflammatory conditions, some of them representing high risk factors for severe COVID-19, including atherosclerosis and cardiovascular diseases. Indeed, an apolipoprotein E-knockout mouse model of dyslipidemia, intraperitoneally treated with quercetin, showed smaller atheromatous lesions, decreased atherosclerosis, and decreased inflammatory lymphocyte and macrophage infiltration through coronary arteries [99]. In addition, in a murine model of rheumatoid arthritis, quercetin inhibited neutrophil infiltration, promoted the apoptosis of activated neutrophils, and reduced plasma levels of inflammatory cytokines, suggesting that quercetin can ameliorate inflammation in rheumatoid arthritis mice by inhibiting neutrophil activities [100]. Furthermore, oral quercetin was able to effectively improve collagen-induced arthritis mice by establishing Th17/Treg balance, inhibiting NLRP3 inflammasome, and reducing inflammatory mediators (TNF-α, IL-1β, IL-6, PGE2, COX-2, and iNOS) through heme oxygenase 1 expression [101]. Then, in line with this study and in vitro studies, other in vivo investigations indicate that the inhibition of inflammasome by quercetin represents an important mechanism underlying its anti-inflammatory activity in several IL-1-mediated inflammatory diseases [102,103,104,105]. For example, in a diabetic murine model, quercetin played a neuroprotecive role for diabetic encephalopathy by inhibiting the NLRP3 inflammasome and thus the production of IL-1β and IL-18 pro-inflammatory cytokines [105].

#### 3.1.3. Quercetin as Immunomodulator in Clinical Trials

The beneficial immunomodulatory activity of quercetin has also been investigated in humans in clinical trials for the prevention and/or treatment of human inflammatory conditions including prostatitis, cystitis, rheumatoid arthritis, and hypercholesterolemia as well as cardiovascular diseases, metabolic syndrome, cancers, and inflammation induced by intensive exercise and respiratory infections [56].

In the majority of studies, contrasting results have been reported concerning quercetin’s capability of inhibiting the production of circulating and/or tissue inflammatory mediators in different human inflammatory conditions. However, the number of subjects enrolled in clinical trials is not enough to draw definitive conclusions, and larger, randomized, placebo-controlled trials are warranted. In two prospective randomized, double-blind, placebo-controlled trials including thirty men with chronic prostatitis syndromes (nonbacterial chronic prostatitis and prostatodynia) and twenty-two patients with interstitial cystitis, oral supplementation of quercetin (500 mg twice daily for 1 month) was well tolerated and provided significant symptomatic improvement in 65% and 99% of patients, respectively [106,107]. Then, treatment with oral supplementation of quercetin (120 mg/daily for two months) in thirty patients with coronary artery disease induced a reduction in serum IL-1β and TNFα levels by decreasing the transcriptional activity of NF-κB [108]. Moreover, oral quercetin supplementation was found to be relatively safe, causing no significant adverse effects; headache and temporary peripheral paresthesia were the only effects observed in two out of thirty patients [108]. In contrast, in a randomized, placebo-controlled, double-blind trial including twenty patients with rheumatoid arthritis, oral supplementation with quercetin and vitamin C (166 mg + 133 mg/capsule; three capsules/day = 900 mg/day for 4 weeks) did not change either the disease severity or serum levels of inflammatory mediators (TNF-α, IL-1β, IL-6, and CRP) [109]. In line with these data, quercetin supplementation at 500 (N = 38) and 1000 (N = 40) mg/d for 12 weeks significantly increased plasma quercetin levels but had no influence on innate immune function or inflammation (IL-6, TNF, leucocyte subset cell counts, NK cell activity, and lymphocyte subsets or phagocytosis) in adult female subjects [110].

Contrasting results also exist on the capability of quercetin ingestion to protect exercise-stressed athletes from exercise-induced muscle inflammation. In a study where sixty-three ultramarathon athletes were randomized to quercetin and placebo groups and ingested 1 g/d quercetin for 3 weeks before a run, quercetin failed to reduce muscle damage, inflammation, plasma cytokine levels, and leucocyte cytokine mRNA expression [111]. In a group of forty male cyclists, quercetin ingestion (1 g/d, 3 weeks before cycling and 3 days during cycling) significantly reduced leucocyte IL-8 and IL-10 mRNA; however, quercetin did not influence muscle inflammation, including NF-κB content, IL-8, and TNF-α mRNA or COX-2 mRNA expression [112].

To assess the anti-inflammatory activity of quercetin in humans, by a meta-analysis of available randomized controlled trials, Mohammadi-Sartang et al. [113] quantified the net effect of quercetin on circulating levels of CRP, which is a very sensitive inflammatory biomarker, showing that high (≥500 mg) daily quercetin intakes significantly lower CRP concentration. Another systematic review and meta-analysis of randomized controlled trials determined that quercetin supplementation (≥500 mg/day for ≥8 weeks) in patients with metabolic syndrome and related disorders significantly reduced fasting plasma glucose levels, suggesting a favorable impact of quercetin on adipose tissue inflammation [114].

Finally, limited clinical evidence suggests that supplemental quercetin may decrease the risk for respiratory infections in humans. In addition, when the beneficial effect of quercetin was present, this was not always associated with the modulation of the host immune response, suggesting that the activity of quercetin against virus respiratory infections might be very likely due to its anti-viral rather than its immunomodulatory activity. In a randomized, double-blinded, placebo-controlled trial conducted in a large community group (N = 1002) of subjects (18–85 years) taking 500 or 1000 mg/day quercetin or a placebo for 12 weeks, it has been observed that the whole group quercetin supplementation had no significant influence on the rates of upper respiratory tract infections compared to placebo. However, in a subgroup of subjects who self-rated themselves as physically fit, 1000 mg/day quercetin resulted in a statistically significant reduction in total sick days and symptom severity associated with upper respiratory tract infections [115]. In another study, quercetin (100 mg/day) did not modulate host immune response (including NK cell activity, PHA-stimulated lymphocyte proliferation, or polymorphonuclear oxidative burst activity), but it significantly reduced the upper respiratory tract infections incidence (one out of 20 subjects in quercetin versus nine out of 20 in the placebo group) in exercise-stressed athletes during the two-week post-exercise period [116]. In line with these observations, under double-blind conditions, 1000 mg of quercetin supplementation three weeks before, during, and two weeks after a three-day period of cycling resulted in a markedly lower incidence of upper respiratory tract infections in the two weeks after the intensified training, but it had no effect on exercise-induced immune dysfunction, inflammation, and oxidative stress [117]. A similar lack of effect on strenuous exercise-induced immune system perturbation (granulocyte respiratory burst, NK cells, neutrophil and monocyte counts) was found in sixty-three athletes who took 1000 mg/day of quercetin for three weeks before, during, and two weeks after a 160 km race; however, in contrast to the previous study, there were no significant changes in the incidence rates of respiratory infections [118].

### 3.2. Polyphenols as Immunomodulators to Combat Severe COVID-19: Quercetin as a Research Model

Since the start of the COVID-19 pandemic, a large body of literature has suggested and recommended the potential use of polyphenols as pharmacological agents to strengthen immunity against SARS-CoV-2 infection and to contrast the rising of “cytokine storm” in severe COVID-19 [50,51,54,55]. Among polyphenols, the use of quercetin has been proposed as a therapeutic tool for COVID-19 to counteract severe inflammation, which is one of the main life-threatening conditions in patients with COVID-19 [52,53]. In addition, quercetin supplementation has been recently approved in clinical trials for COVID-19 prevention and/or therapy (Table 2).

The analysis of the immunomodulatory and anti-inflammatory activity of quercetin in different in vitro models taken into consideration in this review shows that quercetin can affect several aspects of immune response, potentially counteracting most immunological biomarkers of severe COVID-19 (Table 1), including inflammatory cytokine production by innate immune cells, inflammasome and autophagy activation, and inflammatory immune cells recruitment from blood to tissues. However, it should be pointed out that only a few research studies have been focused on the immunomodulatory activity of quercetin in COVID-19-related experimental models, such as respiratory epithelial cells and alveolar macrophages, which are the very first inflammatory cells potentially involved in the pathogenesis of lung inflammation in COVID-19. In addition, no basic research exists on the immunomodulatory activity of quercetin in SARS-CoV-2-infected cell lines, using primary human airway epithelial cells and primary human alveolar macrophages.

The data on the immunomodulatory effects of quercetin in vivo, in different types of inflammatory diseases in animal models, mostly support the results obtained in vitro. However, there is a need for experimental animal models that are currently used in COVID-19 research [119,120]. No less important, some experimental designs, performed in vitro and in vivo, have shown a preventive rather than a therapeutic immunomodulatory activity of quercetin, suggesting that quercetin has the potential capability of maintaining rather than restoring immune homeostasis. This point should be carefully evaluated when a clinical trial is proposed for COVID-19, in that quercetin perhaps should be supplemented very early in the clinical disease course, before the onset of inflammation, and not in the severe disease.

Although encouraging pre-clinical results exist on the immunomodulatory activity of quercetin, results in humans, in clinical trials based on quercetin supplementation in different human inflammatory conditions, do not seem to totally support the data obtained from cells and animals, as the research outcomes are contradictory. Indeed, some clinical trials indicated that quercetin supplementation had no influence on inflammation or immune function [109,110,111,112], although in some cases, there were significantly increased plasma quercetin levels [110]. Other clinical trials produced conflicting results regarding quercetin’s effects on the incidence and/or the duration of upper respiratory tract infections as well as its association with quercetin’s immunomodulatory activity [110,116,117,118]. However, since clinical research with quercetin remains scarce and only small-scale clinical trials have been performed, a larger number of large-scale clinical trials are needed. In addition, since the effect of quercetin appears to be dose- and time-dependent, we should acquire more information regarding the appropriate schedule to be used to obtain immunomodulatory effects in humans. We also think that the activity of quercetin as an immune booster and/or anti-inflammatory agent in humans needs to be further verified with appropriate experimental models.

## 4. Conclusions

Although polyphenols such as quercetin might possess a potential immunomodulatory therapeutic value against SARS-CoV-2-induced immune response dysregulation, further investigation in this specific area is needed to recommend polyphenols as immunomodulatory agents for the treatment of COVID-19.

## Figures and Tables

**Table 1:** Immunological biomarkers of severe COVID-19 as potential targets of immunomodulators.

Innate Immune System	Dysregulated Immune Response
Barrier epithelial cells	Type I IFN response and IFN-stimulated genes NF-κB-dependent pro-inflammatory mediators Cytokines: IL-6, TNF, Il-1β Chemokines: CCL20, CXCL1, 2, 3, 5, 6, 8, 16 STAT signaling NLRP3 inflammasome Immunoproteasome Autophagy
Monocyte/Macrophages	NF-κB-dependent pro-inflammatory mediators Cytokines Chemokines STAT signaling NLRP3 inflammasome Immunoproteasome Autophagy
Neutrophils	Neutrophilia Activation and degranulation NET formation
Eosinophils	Eosinophilia
NK cells	Decreased number Exhausted phenotype
Innate immune receptors	Circulating CRP: increased levels Circulating pentraxins: increased levels Circulating complement fractions: increased levels
**Adaptive Immune System**	**Dysregulated Immune Response**
T Lymphocytes	Lymphopenia Exhausted phenotype Th17/Treg imbalance
B Lymphocytes	Delayed antibody response

**Table 2:** Clinical trials approved for the use of polyphenols for COVID-19.

Compound(s)	Intervention(s)	Title of the Study	Recruitment Status and Source
Cannabidiol	Sublingual oil drops: 3 times a day Phase 1	Synthetic CBD oil droplets as therapy for mild to moderate SARS-CoV-2 infection. Assessment of efficacy and safety	Treatment: not yet recruiting ClinicalTrials.gov: NCT04686539
Curcumin	500 mg curcumin plus 5 mg piperine twice a daily for 2 weeks	Effects of curcumin–piperine co-supplementation on disease duration, severity, and clinical signs, and inflammatory factors in patients with COVID-19: a randomized, double blind, placebo-controlled clinical trial study	Treatment: completed https://www.irct.ir/trial/47529: IRCT20121216011763N46
Curcumin	Sinacurcumin^®^ soft gel: 40 mg; 4 soft gels daily for 2 weeks	Oral nano-curcumin formulation efficacy in management of mild to moderate hospitalized COVID-19 patients: an open-label nonrandomized clinical trial	Treatment: completed https://www.irct.ir/trial/47529: IRCT20200408046990N1
Epigallocatechin-3-gallate	Previfenon: 250 mg Phase 2/3	A multicenter, double-blind, randomized, placebo-controlled clinical trial to protect health workers against COVID-19 by using Previfenon as chemoprophylaxis during a SARS-CoV-2 outbreak. The HERD study	Prevention: not yet recruiting ClinicalTrials.gov: NCT04446065
Resveratrol	Phase 2	Randomized double-blind placebo-controlled proof-of-concept trial of resveratrol, a plant polyphenol, for the outpatient treatment of mild COVID-19	Treatment: terminated. ClinicalTrials.gov: NCT04400890
Quercetin	400 mg/daily Phase 3	A prospective, randomized, open-labeled, controlled trial to study the adjuvant benefits of quercetin phytosome in patients with diagnosis of COVID-19	Treatment: completed ClinicalTrials.gov: NCT04578158
Quercetin	Prophylaxis: 500 mg/daily. Treatment: 1000 mg/daily	The possible effect of quercetin on prophylaxis and treatment of COVID-19	Prevention and treatment: completed ClinicalTrials.gov: NCT04377789
Quercetin	600 mg/daily for 1st week; 400 mg/daily for 2nd week Phase 3	Study to investigate the benefits of dietary supplement quercetin for COVID-19	Treatment: recruiting ClinicalTrials.gov: NCT04861298
Quercetin	1 tablet twice a day Phase 1	Effectiveness of quercetin in the treatment of SARS-CoV2	Treatment: not yet recruiting ClinicalTrials.gov: NCT04853199
